# A Case of Psoas Abscess, Etc.

**Published:** 1890-08

**Authors:** J. L. Cunningham

**Affiliations:** Fort Worth, Texas


### A CASE OF PSOAS ABSCESS, MISTAKEN DURING LIFE FOR HERNIA.

FORT WORTH, TEXAS, May 20th, 1890.

*Editor Daniel's Texas Medical Journal:*

I send for publication the enclosed manuscript. I wish to contribute my share towards tearing off the mask, under which ignorance and pretension are masquerading, in our profession, and show the people what sort of people are posing as doctors.

I think the showing will accentuate and emphasize the necessity of a law regulating the practice of medicine in connection with boards of health, as outlined by the State Association, at its last two meetings. J. L. CUNNINGHAM, M. D.

[The above accompanied the article referred to, and should have been printed in connection therewith.—ED.]

